# Six Recommendations to Build Legitimacy for Translational Research Organizations

**DOI:** 10.3389/fmed.2020.586177

**Published:** 2020-12-08

**Authors:** Christian Rosser, Fritz Sager, Stephen L. Leib

**Affiliations:** ^1^Swiss Institute for Translational and Entrepreneurial Medicine (sitem-insel AG), Bern, Switzerland; ^2^KPM Center for Public Management, University of Bern, Bern, Switzerland; ^3^Institute for Infectious Diseases, University of Bern, Bern, Switzerland

**Keywords:** organizational legitimacy, hybrid organizations, public private partnership, translational research organization, management

## Abstract

Translational research organizations (TROs) face specific challenges to secure resilience and longevity. This perspective article provides the rationale for six hands-on recommendations for the management of legitimacy building in TROs.

## Introduction: Translational Research Organizations in Need for Legitimacy

Translational medicine, defined as the innovation process of transferring the results of basic research into clinical application, “covers a broad range of scientific, regulatory and clinical disciplines” (1). Thus, the success of a translational process in medicine depends on a close integration of stakeholders from industries, clinics, and academia as well as the involvement of the relevant legal bodies and normative authorities (2–4). According to Polese and Capunzo's (5) system theory approach, successful translational medicine is characterized by the fact that numerous actors from both the private and the public sectors benefit from the outcome of joint activities within the same system. Such a system is sustainable only under the condition that intrasystemic relationships lead to results that benefit the actors and therefore allow future resources to be easily transferred to the system.

In line with these findings, both politics and science promote the organizational form of public–private partnership (PPP) for the advancement of translational medicine. Whereas, private organizations aim to implement their strategies effectively and efficiently, public organizations must also do justice to the democratic principles of popular control and participation. One key challenge is therefore how a translational research organization (TRO), organized as a PPP and thereby a hybrid in terms of the dichotomy of economic principles and democracy, may gain legitimacy. In fact, legitimacy is the property that is most important to the sustainable success of a hybrid organization (6). Legitimacy does not exist *per se*. An organization is legitimate only if it enjoys trust as a precondition for the creation of stable relationships among its stakeholders and target population. In the following, we present the Swiss Institute for Translational and Entrepreneurial Medicine (sitem-insel) in Bern, Switzerland, as an empirical case of a TRO (7), before we derive eight types of legitimacy from the literature and apply them to the illustrative case to formulate six hands-on recommendations for TRO practitioners.

## The Sitem-Insel Case

The sitem-insel, located in the Swiss capital, Bern, qualifies as an exemplary PPP: Representatives from private industries and public universities and clinics have in 2014 joined forces to establish the sitem-insel as a non-profit limited company under private law. As part of the same system, these actors expect the sitem-insel to contribute to the growth of the medtech and biotech industries and to thereby generate jobs as well as new products and services in favor of patients. In view of these benefits, shareholders from both the public and the private sectors hold shares worth approximately CHF 12 Mio, while the Swiss Confederation and the canton (state) of Bern subsidized sitem-insel between 2017 and 2020 with approximately CHF 25 Mio each.

With the aim to promote translational medicine, a new building has recently been constructed on the campus of the Bern University Hospital called “Inselspital”—hence the name sitem-insel. The building provides state-of-the-art research laboratory infrastructure on a surface of ~20,000 m^2^ to foster cooperation between partners from the healthcare industry and research groups from hospital clinics and university institutes. They cover various fields including neurology, radiology, anatomy, dental medicine, microbiology, endocrinology, biotherapeutics, and a clinical trials unit. While the sitem-insel is responsible for funding the core and shell of the building, the platforms are funded by renters from the private industry and public organizations. The sitem-insel thereby aims to allow its partners to capitalize on economies of scale and to reduce their translational projects' overall cycle time when bringing innovation to the patient by immersion in the clinical and academic environment.

As a condition for its sustainability, the sitem-insel needs political, financial, and substantive support of its public and private stakeholders. This presupposes sitem-insel's need for legitimacy. In the following, we present several types of legitimacy and illustrate how sitem-insel as a prototypical case of a TRO can make use of them.

## Types of Organizational Legitimacy

According to Suchman (8), legitimacy is “a generalized perception or assumption, that the actions of an entity are desirable, proper, or appropriate within some socially constructed system of norms, beliefs, and definitions.” Distinguishing the three categories of pragmatic, moral, and cognitive legitimacy, one can discern eight mutually overlapping and reinforcing legitimacy types.

### Pragmatic Legitimacy

The basis of pragmatic legitimacy is the self-interest that an organization's instrumental value promises to satisfy. Specific policies, services, and goods have an instrumental value that organizations may trade with their audiences. This is what Suchman calls exchange legitimacy. At a higher level, Suchman (8) speaks of influence legitimacy when the organization is perceived to respond to the larger interest.

### Moral Legitimacy

Moral legitimacy is based on normative evaluation, resting on the congruence between collectively held norms and an organization's achievements. First, consequential legitimacy regards an organization's achievements in terms of “consequential effectiveness” (8). Second, procedural legitimacy stems from an organization's practices perceived to be sound and professional. Third, structural legitimacy asks whether an organization promises to be the “right organization for the job.” Finally, an organization's personal legitimacy stems from the charisma, credibility, and appeal of its leaders as “moral entrepreneurs” (8).

### Cognitive Legitimacy

Cognitive legitimacy denotes whether an organization's role in the environment finds acceptance. When its environment deems an organization's goals desirable, Suchman speaks of comprehensibility legitimacy. An organization may also just be “taken for granted” (8). Such taken-for-granted legitimacy does not depend on evaluation. It therefore is the most powerful form of legitimacy. However, at the same time, it also is extremely rare.

## How to Get There: Legitimacy Building for the TRO

Our framework offers different strategies for legitimacy building in TROs that we illustrate with the sitem-insel case as a prototypical case.

### How to Build Pragmatic Legitimacy

The TRO should secure long-term, process-oriented stakeholder involvement to build *exchange legitimacy* and *influence legitimacy*. As PPPs are accountable to both market demands and state institutions, they assemble stakeholders with heterogeneous interests. This diversity may lead stakeholders to seek the lowest common denominator, which may differ from the intentions of individual stakeholders. Managers of TROs should therefore flexibly cooperate with stakeholders and acknowledge their different ambitions. The sitem-insel must know its clients, constituencies, and members to protect their interests. It should include relevant actors, such as political incumbents and administrative officeholders, private industry representatives, experts, and members of the civil society in decision-making processes and implementation. Exchange platforms can support its diverse stakeholders in the deliberation of strategic objectives. This inclusion is an investment into the implementation of these objectives as it leads to reliable expectations and establishes binding rules for the interaction among actors.

As sitem-insel aims to establish an innovation hub for translational medicine in the long run, priority must be assigned to the inclusion of powerful stakeholders. First, the support of the relevant legal bodies and normative authorities must be ensured. The sitem-insel apparently has this support, which materialized in subsidies of approximately CHF 50 Mio. The second priority of stakeholder inclusion lies with stakeholders as investors, before, third, less decisive players should have the opportunity to participate as well. This process may be oriented toward conformism, i.e., the aim of following “dictates of preexisting audiences within the organization's current environment” (8). Accordingly, the involvement of the Inselspital's head of clinics as the main shareholder was an important basic decision for the sustainable development of the sitem-insel, quite simply because the clinical physicians are important drivers of translation in medicine. In addition, the sitem-insel may concentrate on cooperation with Bern-based partners such as local companies and institutes of the university, before extending the network to national and international levels.

### How to Build Moral Legitimacy

*Consequential legitimacy* has to do with the TRO's instrumental performance. Pozen and Kline (9) suggest several measurable aims TRO's must achieve to perform in compliance with their audience's expectations. These aims include funding and commercial investment, size and quality of the organization's staff, talent turnover, number and importance of new collaborations, the volume of pipeline (i.e., new projects) and its progress, the number of patents and publications, and the knowledge transfer of innovation. Accordingly, the sitem-insel's key performance indicators measure not only output performance but also the organization's impact.

*Personal legitimacy* refers to the support for an organization's leaders because of their credibility and appeal. In this context, the sitem-insel needs staff at the strategic level, from both the public and the private sectors, who assume the role of legitimization promoters. For instance, it makes sense to involve the head of clinics, directors of university institutes, and senior executives from the local industry in the board of directors and advisory bodies. Due to their high hierarchical rank and reputation, they can justify innovation processes, acquire the necessary resources, and overcome the resiliency of change. There are not many institutes of translational medicine in Switzerland. Consequently, sitem-insel's management board should additionally rely on international experience in promoting translational medicine.

*Procedural legitimacy* stems from sound administrative practices and professional routines. Accordingly, the sitem-insel must not only incorporate substantive medical knowhow. Including financial and entrepreneurial expertise is equally important. In fact, sound financial programs as well as unambiguous reporting and controlling processes are often lacking within entrepreneurial public programs, even though they are crucial for the performance of TROs (10). In this context, for instance, it may be reasonable for the sitem-insel to outsource audit and assurance, tax and legal, or financial advisory to professional service firms.

*Structural legitimacy* has to do with the support of organizational capacity. Most importantly, silo building must be avoided. It therefore makes sense for the sitem-insel to adopt a “flat and flexible structure wherein different departments are interconnected” (11). Accordingly, a matrix organization may serve as the appropriate knowledge-creating organizational structure. Because innovation benefits from the inclusion of stakeholders as they “realize better, more innovative solutions by harnessing each other's knowledge and expertise” (12), open innovation platforms may be added to the TRO's portfolio. In general, the sitem-insel may perform mimetic isomorphism or, in other words, imitate processes and structures of successful TRO's to promote translational and entrepreneurial medicine. For instance, given the Inselspital's powerful brand name in Switzerland's medical landscape, it was important to incorporate the term “insel” into sitem-insel's brand name. The physical proximity of sitem-insel's new building to the university hospital is a major asset beyond branding, as the Inselspital campus comprises all relevant tertiary medical disciplines.

### How to Build Cognitive Legitimacy

*Comprehensibility legitimacy* refers to the support a TRO enjoys thanks to its compliance with the cognitive scripts and belief systems of its audience. As hybrid organizations, TROs need to respond to various normative reference systems such the state, society, and the market. All discussed strategies add to gaining comprehensibility legitimacy. Finally, *taken-for-granted legitimacy* goes beyond managerial control. It is a function of all other forms of legitimacy.

## Conclusion

TROs organized as PPPs are considered by stakeholders of both the public and the private sectors as part of a long-term solution to issues affecting society and the economy at large. As TROs cannot rest upon a single reference system like the market or the state alone, organizational legitimacy is a precondition for their sustainable success. However, legitimacy must be built. The presented strategies provide practical guidance to do so. Our recommendations, summarized in Table 1, may help TROs to fulfill their vital role for the industry, academic medicine, and health policy.

**Table 1:** Six recommendations for the management of legitimacy building in TROs.

**Legitimacy category**	**Legitimacy type**	**Explanation**	**Recommendation**
Pragmatic	Influence and exchange	Support for an organization in anticipation of its responsiveness to its audience's interest	1) Secure long-term, process-oriented stakeholder involvement
Moral	Consequential	Support for an organization because of the level of reward of its policies	2) Serve instrumental demands by performance reputation strategy
	Personal	Support for an organization's leaders because of their credibility and appeal	3) Involve top staff at the control level and secure management turnover
	Procedural	Support for an organization's sound administrative practices and professional routines	4) Secure expertise in financial management and business administration
	Structural	Support for capacity of organizational structure: Does the organization promise to work in favor of the public?	5) Secure flat and flexible organizational structures
Cognitive	Comprehensibility	Support for an organization's compliance with cognitive scripts, belief systems, and perceived reality	6) Imitate successful hybrid organizations

## Data Availability Statement

The original contributions presented in the study are included in the article/supplementary material, further inquiries can be directed to the corresponding author/s.

## Author Contributions

CR, FS, and SL have jointly conceptualized, drafted, written, read, and approved the final manuscript. All authors contributed to the article and approved the submitted version.

## Conflict of Interest

The authors declare that the research was conducted in the absence of any commercial or financial relationships that could be construed as a potential conflict of interest.
